# Collagen Fiber-Oriented Pullout Repair for Medial Meniscus Posterior Root Tear of the Knee

**DOI:** 10.1016/j.eats.2025.103786

**Published:** 2025-07-30

**Authors:** Seikai Toyooka, Takumi Nakagawa, Keisuke Kita

**Affiliations:** a Department of Orthopaedic Surgery, Teikyo University School of Medicine, Tokyo, Japan; b Department of Othropaedic Sports Medicine, JCHO Osaka Hospital, Osaka, Japan

## Abstract

The medial meniscal posterior root tear (MMPRT) is a medial meniscal injury that is common in middle-aged and elderly patients. In many cases, MMPRTs require repair, since they result in the loss of circumferential hoop stress and are highly correlated with knee generation. Although clinical results of MMPRT repair have improved, poor results can occur if the tunnel position or reduction method is suboptimal. There are many reports on how to secure the meniscus, but there are few reports on how to create the bone tunnel. Here, we describe a method for creating a bone tunnel through a posteromedial portal for MMPRT repair. This repair can be performed along the fiber direction of the meniscus, offering the potential for more physiological fixation. In addition, the bone tunnel can be created in a more anatomically accurate position because the footprint is more visible than with conventional methods, without the need for special instruments.

The medial meniscus posterior root tear (MMPRT) is one of the most common meniscal injuries in middle-aged and elderly patients and should be repaired whenever possible.^1^, ^2^, ^3^ MMPRT repair not only improves patient outcomes by restoring the meniscus hoop stress function but also reduces the risk of radiographic knee osteoarthritis (OA) and the need for knee arthroplasty.^4^^,^^5^ The position of the bone tunnel for the meniscus to be pulled through and the method of meniscal reduction are very important in this surgery.^6^ Poor positioning of the bony tunnel to restore the meniscus results in poor mechanical properties.^7^ Moreover, changes in the morphology of the meniscus due to improper reduction result in poorer postoperative outcomes.^6^^,^^8^ There are many reports on how to secure the meniscus in relation to MMPRT repair; however, there are few reports on how to create the bony tunnel. Generally, the target guide is inserted through the anterior portal, and a bone tunnel is created from the distal medial aspect of the tibia. However, at our institution, we directly insert a guide pin through the posteromedial portal and create a bone tunnel that exits lateral to the tibial tuberosity for several reasons. The biggest advantage of this method is that the tunnel direction can be created in the same direction as the posterior meniscal fiber, making hoop reproduction more physiologically possible. The second advantage is that it facilitates confirmation of the footprint of the medial meniscal posterior horn. Typically, when the camera and target guide are inserted into the narrow intercondylar space, the position of the footprint is not clearly visible. With this method, only the camera enters the intercondylar space from the anterior portal, allowing for manipulation while confirming the anatomic position. The third advantage is that expensive target guides or special guides are not required. By using this method, improvements in the outcomes of MMPRT repair are expected in the future. This study was approved by the Ethics Committee of the Graduate School of Medicine, Teikyo University. Informed consent was obtained from all participants.

## Surgical Technique

A video presenting this technique is provided (Video 1).

### Surgical Indications

The ideal patients for MMPRT repair are individuals who meet the following criteria: (1) patients who received treatment early after injury, preferably within 3 months; (2) patients who are relatively young and active; (3) patients who do not have advanced knee OA; (4) patients who do not have severe varus knee alignment abnormalities; (5) a low body mass index; and (6) the ability to comply with a postoperative rehabilitation program.

### Patient Positioning and Diagnostic Arthroscopy

Under general or spinal anesthesia, the patient is placed on a radiolucent operating table on their back. A tourniquet is then applied, and a support plate is attached to the proximal femur to apply valgus stress. Surgery with the lower limbs hanging down is not recommended. When the posteromedial portal is used, hanging the lower limbs down will cause the joint capsule to not expand, and the posteromedial view will be poor. An arthroscopic examination is performed to assess meniscus injury and knee OA. A 45° or greater oblique mirror is recommended for viewing the attachment point of the MMPRT through the anterior portal.

### Technique

In cases where there is advanced OA or a medial proximal tibial angle alignment abnormality of less than 85°, the surgical technique is often used in combination with high tibial osteotomy. In such cases, the medial collateral ligament (MCL) is detached distally to the osteotomy site. The MCL procedure facilitates arthroscopic examination of the medial compartment and MMPRT. If the high tibial osteotomy is not used in combination, the MCL sometimes requires the pie-crusting technique.

**Fig 1:**
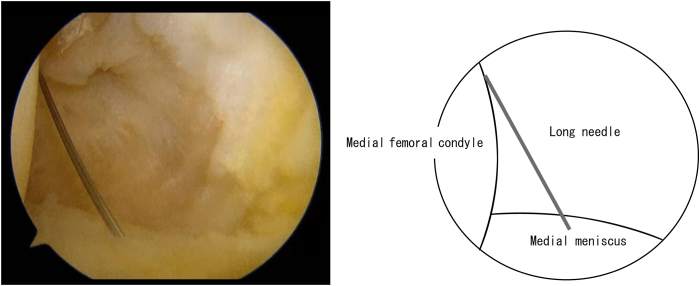
Needle insertion. With the left knee flexed at 90°, the posteromedial compartment is visualized via the anteromedial portal, between the posterior cruciate ligament (PCL) and medial femoral condyle. A long needle is inserted as posteriorly as possible, aimed toward the tibial attachment of the PCL, to confirm the position and direction of the posteromedial portal.

**Fig 2:**
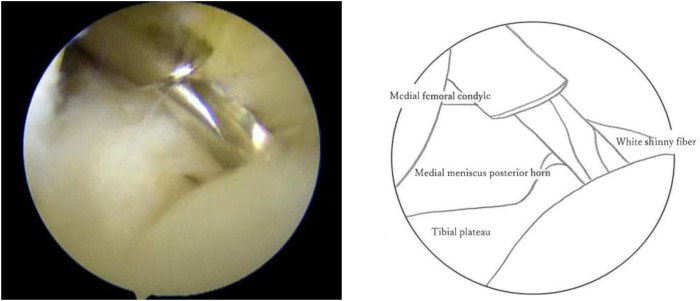
Guidewire insertion. With the left knee flexed at 90°, the posteromedial compartment is visualized via the anteromedial portal, between the posterior cruciate ligament and medial femoral condyle. The portal is enlarged, and a thin cannula or one from the Henning inside-out meniscal repair system is inserted, followed by a 2.4-mm drill-tip guidewire. The posterior root footprint is confirmed via the anterior portal, typically visualized as a white, shiny fiber anterior and medial to the posterior cruciate ligament. When the guide pin is correctly positioned, it exits slightly lateral to the tibial tuberosity.

**Fig 3:**
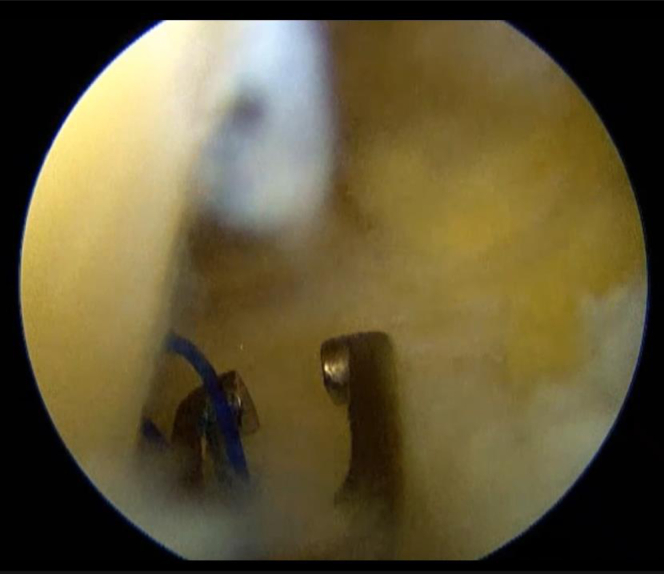
Retrieval of the suture. With the left knee flexed at 90°, the posteromedial compartment is visualized via the anteromedial portal, between the posterior cruciate ligament and medial femoral condyle. A skin incision is made at the guide pin exit, and a suture-retrieval device with a thread is passed through the tunnel from this point. Then, the thread is retrieved to the anterior portal. Using a suture relay technique with this thread, the suture thread passed through the medial meniscus is pulled into the bony tunnel.

**Fig 4:**
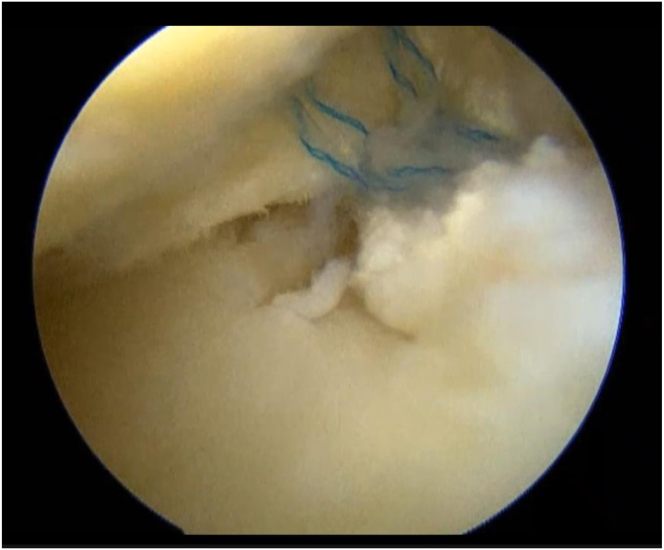
Suture fixation. With the left knee flexed at 90°, the posteromedial compartment is visualized via the anteromedial portal. Fix these sutures to the tibial cortex using any fixation device, such as an EndoButton (Smith & Nephew), with the knee joint flexed at 30°.

The knee joint is held in a 90° flexion position, and the posterior medial compartment is viewed through the anterior medial portal, between the posterior cruciate ligament (PCL) and the medial femoral condyle. The position of the medial saphenous vein is confirmed from the medial surface of the knee joint. While the posteromedial portal of the knee joint is created, special attention should be given to the saphenous nerve surrounding the saphenous vein. First, insert a long needle to confirm the position and direction of the posteromedial portal (Fig 1). Insert the needle as posterior as possible and confirm that it is directed toward the tibial attachment of the PCL. After the portal is enlarged, a thin cannula or a cannula of the Henning system for inside-out meniscal repair (Stryker Japan) is inserted, followed by a 2.4-mm Drill-tip Guide Wire (Smith & Nephew) that is advanced into the cannula. The footprint of the posterior root is confirmed from the anterior portal. A white shiny fiber is usually observed slightly anterior and medial to the PCL (Fig 2). If the guide pin is inserted directly into that position, the pin coming out from the area slightly lateral to the tibial tuberosity can be observed. The medial meniscus posterior segment or posterior horn is subsequently secured via any method of choice and pulled into the bony tunnel. If further stabilization of the entire meniscus is desired, the circumferential fibro-augmented meniscectomy method described by Kita et al.^9^ can be used. In this case, pull the thread at the posterior horn into the bone tunnel as well. Make an incision in the skin around the guide pin exit and insert a suture-retrieval device with a thread that is passed through the tunnel from the pin exit. Secure the thread within the joint, and pull the thread into the bone tunnel using the suture relay technique (Fig 3). Fix these sutures to the tibial cortex using any fixation device, such as an EndoButton (Smith & Nephew), with the knee joint flexed at 30° (Fig 4).

### Postoperative Rehabilitation

The patient begins walking with crutches and initially does not put any weight on the leg. Partial weightbearing is started from the second week after surgery, and full weightbearing is allowed from 4 weeks after surgery. After surgery, the patient is kept in an extended position using a brace for 2 weeks, and range-of-motion exercises are started 2 weeks after surgery. Full range-of-motion exercises are possible after 4 weeks. The patient can return to normal activities after 3 months.

## Discussion

Here, we described a bone tunnel creation technique for MMPRT repair. The primary object of this surgery is to restore the hoop stress function of the meniscus.^3^^,^^10^ In this respect, the direction of traction also affects the improvement of the hoop stress effect of the meniscus.^11^^,^^12^ Conventional bone tunnels create the exit of the bone tunnel on the medial side of the distal tibia. In this case, a 90° or greater “killer turn” occurs in the bone tunnel, and it is unclear whether the pulling force is adequately transmitted to the meniscus. In the future, bone tunnel enlargement and thread wear may also be considered. In the method described here, the bone tunnel is directed toward the lateral aspect of the tibial tuberosity. It is possible to pull in the same direction as the fiber direction of the medial meniscus posterior horn, thereby maximizing the meniscal hoop effect. It may be possible to apply more physiological traction than before.

In addition, to successfully perform MMPRT repair, it is necessary to create bone tunnels in anatomically accurate locations. In conventional MMPRT repair, a target guide for creating a bone tunnel is inserted into the narrow intercondylar space at the same time as an arthroscopic camera. It is technically difficult to create a bone tunnel while confirming the accurate footprint position of the posterior horn of the meniscus since the guide comes in front of the camera. It is sometimes necessary to determine the position of the bone tunnel via a blind technique. With the method described here, the footprint can be viewed directly to create the bone tunnel from the posteromedial portal without using a guide. Improved visibility is likely to help create accurate bone tunnel locations.

Finally, the method described in this article does not require a special target guide. In most cases, the MMPRT bone tunnel is created via an anterior cruciate ligament tibial guide or an MMPRT guide through the anterior portal. The area around the posterior horn of the medial meniscus is particularly narrow in small women and muscular men. In these patients, even if the MCL is relaxed, the guide may not reach the footprint sufficiently. If the guide does not reach a sufficient distance into the footprint, a bone tunnel may be created in the weightbearing surface of the articular cartilage. This is likely to lead to postoperative pain.^13^ Excessive loosening of the MCL can lead to MCL insufficiency, which can cause knee instability and pain after surgery.^14^^,^^15^ Excessive treatment of the MCL should be avoided. The method described here has many advantages, summarized in Table 1. By paying attention to several points summarized in Table 2, it has the potential to improve the treatment outcomes of MMPRT repair.

**Table 1:** Advantages and Disadvantages of the Bone Tunnel Creation Through the Posteromedial Portal for Medial Meniscus Posterior Root Repair

Advantages	Disadvantages
Creation of tunnels in the direction of the fibers of the posterior horn of the medial meniscus may lead to improved hoop effect.	The difficulty in creating a posteromedial portal in an obese person
Improved visibility of the posterior horn of the medial meniscus allows for the creation of a bone tunnel in anatomically accurate positions.	The difficulty in creating a posteromedial portal in a synovial inflammation
No special guide is required for bone tunnel creation.	Risk of injury to the sciatic nerve when creating a posteromedial portal
There is no need to loosen the medial collateral ligament when securing the meniscus from the posteromedial portal.

**Table 2:** Pearls and Pitfalls of the Bone Tunnel Creation Through the Posteromedial Portal for Medial Meniscus Posterior Root Repair

Pearls	Pitfalls
The direction of the bone tunnel is determined by the position of the posteromedial portal. If created anteriorly, it will hit the medial posterior condyle of the femur, and the footprint cannot be targeted. Therefore, it should be created as posteriorly as possible.	If the surgeon does not use a cannula when inserting the guide pin, the soft tissue will get tangled.
When using the high tibial osteotomy, the bone tunnel exit must be created proximally to the osteotomy site. Therefore, the posteromedial portal must be created distally.	

## Disclosures

All authors (S.T., T.N., K.K.) declare that they have no known competing financial interests or personal relationships that could have appeared to influence the work reported in this paper.

## Supplementary Data

Video 1 Description of the surgical technique: this video shows a surgical technique to create a bone tunnel from the posteromedial portal when performing a pullout repair for medial meniscus posterior root tear. By creating the bone tunnel in the same direction as the meniscal fibers, traction can be applied, enabling a physiological repair that replicates the meniscus’s hoop effect. Improved visibility of the posterior horn of the medial meniscus allows for the creation of a bone tunnel in an anatomically accurate location. No special guides are required for bone tunnel creation. This method has the potential to improve surgical outcomes.
